# Stacking engineering induced Z-scheme MoSSe/WSSe heterostructure for photocatalytic water splitting

**DOI:** 10.3389/fchem.2024.1425306

**Published:** 2024-06-28

**Authors:** Longjun Ren, Zongfa Liu, Zhen Ma, Kai Ren, Zhen Cui, Weihua Mu

**Affiliations:** ^1^ School of Mechanical Engineering, Wanjiang University of Technology, Maanshan, China; ^2^ School of Automotive Engineering, Weifang Vocational College, Weifang, China; ^3^ School of Agricultural Engineering, Jiangsu University, Zhenjiang, China; ^4^ School of Mechanical and Electronic Engineering, Nanjing Forestry University, Nanjing, China; ^5^ Medical Oncology, Luoyang Central Hospital, Luoyang, China; ^6^ School of Automation and Information Engineering, Xi’an University of Technology, Xi’an, Shaanxi, China; ^7^ Wenzhou Institute, University of Chinese Academy of Sciences, Wenzhou, China

**Keywords:** first-principle calculations, heterostructure, stacking, Z-scheme, photocatalyst

## Abstract

Stacking engineering is a popular method to tune the performance of two-dimensional materials for advanced applications. In this work, Jansu MoSSe and WSSe monolayers are constructed as a van der Waals (vdWs) heterostructure by different stacking configurations. Using first-principle calculations, all the relaxed stacking configurations of the MoSSe/WSSe heterostructure present semiconductor properties while the direct type-II band structure can be obtained. Importantly, the Z-scheme charge transfer mode also can be addressed by band alignment, which shows the MoSSe/WSSe heterostructure is an efficient potential photocatalyst for water splitting. In addition, the built-in electric field of the MoSSe/WSSe vdWs heterostructure can be enhanced by the S–Se interface due to further asymmetric structures, which also results in considerable charge transfer comparing with the MoSSe/WSSe vdWs heterostructure built by the S–S interface. Furthermore, the excellent optical performances of the MoSSe/WSSe heterostructure with different stacking configurations are obtained. Our results provide a theoretical guidance for the design and control of the two-dimensional heterostructure as photocatalysts through structural stacking.

## Introduction

Recently, hydrogen has been considered the most environmental friendly energy source because the products of combustion are mainly water. At the same time, two-dimensional (2D) materials have also been widely investigated, after the development of graphene (Geim and Novoselov, 2007). Graphene shows ultrahigh electrical and thermal conductivity attributed from the unique electronic properties (Miro et al., 2014). In order to make up for the application limitations by zero bandgap in graphene, other 2D materials are also gradually reported. Phosphorene can be prepared by electrochemical exfoliation (Ambrosi et al., 2017), which is a promising field-effect transistor (Li et al., 2014), showing charge-carrier mobility as high as 1,000 cm^−2^V^−1^s^−1^. Blue phosphorus can be obtained by the epitaxial growth method, and the bandgap is measured as 1.10 eV using the scanning tunneling spectroscopy method (Zhang et al., 2016). The transition metal dichalcogenides (TMDs) are also popular that the excellent optical, electronic, and catalytic performances present the potential applications as a photocatalyst (Ren et al., 2019a; Rao et al., 2021) and nanodevice (Cui et al., 2015). In particular, Janus TMDs are famous for the interesting properties brought about by asymmetric structures, which can be prepared based on chemical vapor deposition (Lu et al., 2017). Such structural symmetry-breaking results in a built-in electric field and obtained ultrafast charge separation (Liang et al., 2018). The Janus MoSSe presents excellent thermal performances, which can also be tuned by the defect (Qin et al., 2022). The novel optical and electronic properties of the Janus MoSSe show the potential applications as photocatalytic and photovoltaic devices (Li et al., 2017; Ren et al., 2020a; Liu et al., 2021; Sun et al., 2022).

To explore more 2D materials as photocatalyst for water splitting, the methods based on large-scale searches of material structures and elements are developed (Tang et al., 2019; Sun et al., 2021; Xu et al., 2021; Ren et al., 2022a; Ren et al., 2022b). For example, the Janus B_2_P_6_ monolayer is proposed as an excellent photocatalyst by novel built-in electric field and the solar-to-hydrogen efficiency (Sun and Schwingenschlögl, 2020); the B_2_P_6_ monolayer also presents tunable electronic properties under the external strain (Ren et al., 2021a) and atomic adsorption (Ren et al., 2022c). XN (X = C, Si, Ge, and Sn) monolayers are predicted possessing decent mechanical and catalytic properties, in particular, the SnN monolayer shows ultrahigh carrier mobility as large as 1.55 × 104 cm^2^·V^−1^·s^−1^ (Ren et al., 2023a). Using the 2D van der Waals (vdWs) heterostructure as a photocatalyst to decompose water is more advantageous than the monolayer because the photogenerated electrons and holes can be separated into different layers for H_2_ (reduction reaction) and O_2_ (oxidation reaction) (Zhang et al., 2023). Furthermore, the catalytic performance and optical properties of the vdWs heterostructure obviously depends on the external strain (Guo et al., 2020) and stacking configurations (He et al., 2014; Ren et al., 2022d). In particular, a heterostructure with a Z-scheme photocatalytic mechanism has received some attention because of its unique photogenerated charge transport pathways (Xu et al., 2018; Tang et al., 2022). For example, the C_3_N_4_/W_18_O_49_ heterostructure was prepared, which presents a switch from the type-II to Z-scheme photocatalyst with a H_2_ evolution rate of 8,597 μmolh^−1^g^−1^ (Huang et al., 2017). The black phosphorus/BiVO_4_ heterostructure also possesses an artificial Z-scheme photocatalytic system with an H_2_ rate of approximately 160 μmolh^−1^g^−1^ (Zhu et al., 2018). Theoretically, some promising 2D Z-scheme heterostructures used as the photocatalyst are proposed, such as PtS_2_/arsenene (Ren et al., 2020b), CdO/HfS_2_ (Zhang Q. et al., 2022), C_7_N_6_/Sc_2_CCl_2_ (Meng et al., 2022), and BCN/C_2_N (Zhang et al., 2018) etc. For a heterostructure based on Janus TMDs, the asymmetric structure also induces the uneven force in the Janus heterostructure (Ren et al., 2023b), and a naturally curved interface enhances tensile strength because the external strain first needs to overcome intrinsic deformation. In addition, such an intrinsic curved interface also suppressed the heat transport capacity (Ren et al., 2022e), which explains the MoSSe/WSSe heterostructure can be used as thermal management in nanodevice. Therefore, the MoSSe/WSSe vdWs heterostructure tuned by stacking means as a photocatalyst is meaningful for further exploration. In addition, stacking engineering is feasible in experiments for the Janus TMD heterostructure (Zhang et al., 2020), and some investigations also show the tunable electronic and optical properties by the stacking method (Xu et al., 2013; He et al., 2014; Shu et al., 2016).

In this investigation, the heterostructure is constructed by MoSSe and WSSe monolayers. The S–Se and S–S interfaces are fully considered to investigate the structural and electronic properties using the first-principles method. The band energy and the flow path of photogenerated charges of the MoSSe/WSSe vdWs heterostructure with different stacking styles are addressed in detail. Then, the dependence on the stacking configuration for the MoSSe/WSSe vdWs heterostructure of light absorption is also obtained.

## Calculation models and methods

In this simulations, all the first-principle calculations are considered by density functional theory (DFT) (Kresse and Furthmüller, 1996a; Grimme et al., 2010), using the Vienna *ab initio* simulation package (VASP) (Kresse and Furthmüller, 1996b) and the Device Studio [Hongzhiwei Technology, Device Studio, Version 2021A, China, 2021, available online at: https://iresearch.net.cn/cloudSoftware, accessed on 2 June 2023] program, which provides a number of functions for performing visualization, modeling, and simulation. DS-PAW software is integrated into the Device Studio program to calculate the electronic properties of the studied system (Blöchl, 1994). The projector augmented wave (PAW) potentials (Kresse and Furthmüller, 1996b) were employed by the generalized gradient approximation (GGA) to describe the core electrons (Perdew et al., 1996). The Perdew–Burke–Ernzerhof (PBE) functional was also conducted to express the exchange correlation functional. The DFT-D3 method was utilized to describe the weak dispersion forces in the vdWs heterostructure by Grimme (Grimme et al., 2010). To obtain decent optical and electronic properties, the Heyd–Scuseria–Ernzerhof hybrid method is addressed (Heyd et al., 2005). In the first Brillouin zone, 17 × 17 × 1 Monkhorst-Pack *k*-point grids were explored with the energy cut-off of 550 eV. The vacuum thickness is set as 25 Å to minimize the interaction between nearby layers. The convergence for force is controlled in 0.01 eV Å^−1^. The energy of the system is chosen by 0.01 meV.

## Results and discussion

**FIGURE 1:**
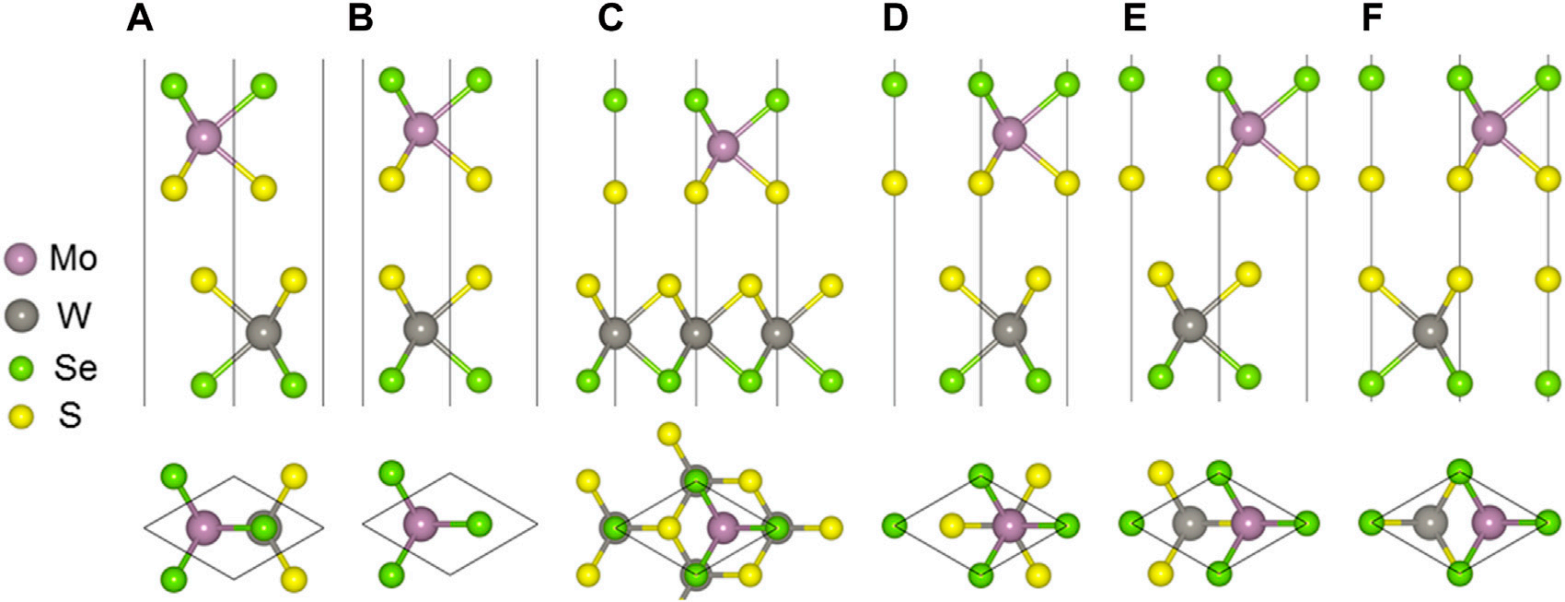
MoSSe/WSSe vdWs heterostructure with **(A)** SS-1, **(B)** SS-2, **(C)** SS-3, **(D)** SS-4, **(E)** SS-5, and **(F)** SS-6 stacking configuration.

The MoSSe and WSSe monolayers are optimized with the lattice constant of approximately 3.228 Å and 3.269 Å, respectively. The band structure of the MoSSe and WSSe monolayers are also obtained in Supplementary Figure S1 using HSE06 calculations. The MoSSe and WSSe monolayers present a semiconductor property and a direct bandgap of approximately 2.100 eV and 2.077 eV, which is in agreement with the reported investigation (Lou et al., 2021). Then, the MoSSe/WSSe heterostructure can be obtained by a lattice mismatch as small as about 1.26%. The MoSSe/WSSe heterostructure is constructed by considering the high symmetry, which can be summarized as six different configurations with the interface formed by S and S atoms. As shown in Figure 1, all the different stacking configurations are maned as SS-1 to SS-6. Similarly, these six stacking configurations can also be reserved while the interface is constructed by S and Se atoms, namely, SSe-1 to SSe-6. All the binding energy (*E*_binding_) is calculated as Eq. 1:

$$E_{\text{binding}}=\left(E_{\text{Heterostructure}}\;–\;E_{\text{MoSSe}}\;–\;E_{\text{WSSe}}\right)/S,$$ (1)

where the *E*_Heterostructure_, *E*_MoSSe_, *E*_WSSe_, and S represent the total energy of the MoSSe/WSSe heterostructure, pure MoSSe, WSSe monolayers, and the area of the system, respectively. All the binding energy of the MoSSe/WSSe heterostructure is summarized in Table 1; one can see that all the lowest binding energy of these 12 MoSSe/WSSe heterostructures is calculated as −34.788 meV/Å^2^ for the SSe-2 stacking configuration. In addition, others are ranging from −25.395 to −34.788 meV/Å^2^, which is also lower than that of graphene (Chen et al., 2013), explaining all these MoSSe/WSSe heterostructures are formed by vdWs forces. Furthermore, the phonon dispersions of the MoSSe/WSSe vdWs heterostructure are calculated as Supplementary Figure S2. One can see that no imaginary frequency exists in the phonon dispersions of the MoSSe/WSSe vdWs heterostructure with SS-1 and SSe-1 stacking configurations, suggesting a dynamic stability of the MoSSe/WSSe vdWs heterostructure.

**TABLE 1:** Calculated binding energy (*E*_*binding*_, meV/Å^2^), the bond length (L, Å), the distance of interface (D, Å), and the bandgap (Eg, eV) of the MoSSe/WSSe heterostructure constructed by different stacking configurations.

	*E*_binding_	*L*_Mo-S_	*L*_Mo-Se_	*L*_W-S_	*L*_W-Se_	*D*	*Eg*
MoSSe		2.415	2.531				2.100
WSSe				2.428	2.543		2.130
SS-1	−33.034	2.411	2.529	2.416	2.534	3.017	1.461
SS-2	−25.395	2.412	2.529	2.417	2.534	3.569	1.693
SS-3	−33.321	2.411	2.529	2.416	2.534	2.971	1.434
SS-4	−31.896	2.411	2.529	2.416	2.534	3.050	1.459
SS-5	−33.091	2.411	2.528	2.416	2.534	2.977	1.390
SS-6	−25.790	2.412	2.529	2.417	2.534	3.581	1.696
SSe-1	−26.439	2.412	2.529	2.418	2.533	3.684	1.474
SSe-2	−34.788	2.411	2.529	2.418	2.532	3.034	1.390
SSe-3	−32.637	2.411	2.529	2.418	2.532	3.178	1.383
SSe-4	−33.966	2.411	2.528	2.418	2.532	3.080	1.319
SSe-5	−34.753	2.412	2.528	2.418	2.533	3.078	1.365
SSe-6	−26.861	2.411	2.529	2.418	2.533	3.608	1.448

The original bond lengths of Mo-S, Mo-Se, and W-S, W-Se are obtained by 2.415 Å, 2.531 Å and 2.428 Å, and 2.543 Å, respectively, in MoSSe and WSSe monolayers. When MoSSe and WSSe are built as an heterostructure, all the Mo-S, Mo-Se, and W-S, W-Se bonds in MoSSe and WSSe can be compressed induced by the vdWs interactions, shown as Table. 1. In addition, the distance of the interface of the MoSSe/WSSe vdWs heterostructures is optimized at approximately 2.971–3.684 Å, which is comparable with the reported vdWs heterostructure, such as CdO/HfS_2_ (Zhang Q. et al., 2022) and MoTe_2_/PtS_2_ (Zhang L. et al., 2022).

**FIGURE 2:**
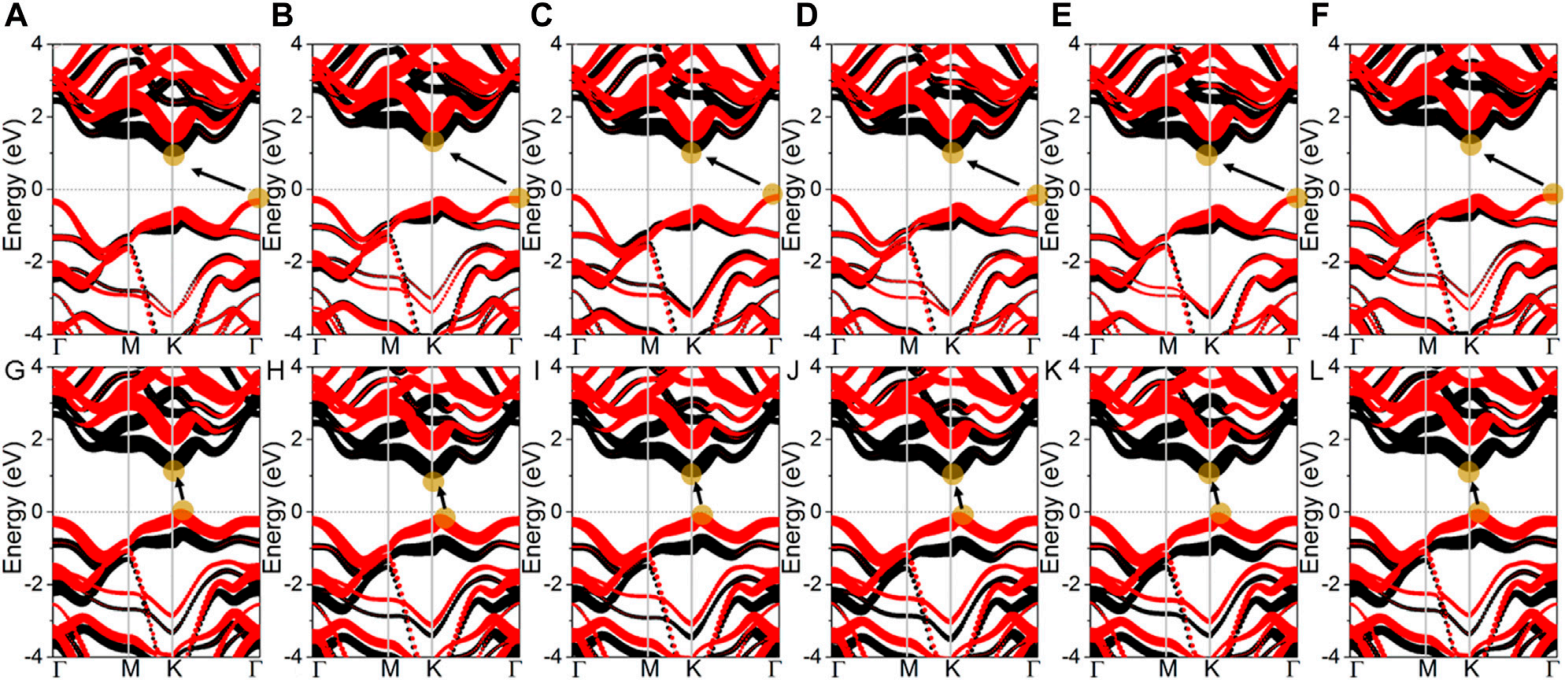
Projected band structure of the MoSSe/WSSe vdWs heterostructure with **(A)** SS-1, **(B)** SS-2, **(C)** SS-3, **(D)** SS-4, **(E)** SS-5, **(F)** SS-6, **(G)** SSe-1, **(H)** SSe-2, **(I)** SSe-3, **(J)** SSe-4, **(K)** SSe-5, and **(L)** SSe-6 stacking configurations. Fermi level is 0; black and red marks are MoSSe and WSSe monolayers, respectively, calculated by DS-PAW.

The projected band structure of the MoSSe/WSSe vdWs heterostructure different stacking styles are calculated as Figure 2. One can see that all these heterostructures present semiconductor characteristics, and the bandgaps are obtained as Table. 1. It is worth noting that the MoSSe/WSSe vdWs heterostructure constructed by the S–S interface show an indirect bandgap with the conduction band minimum (CBM) located at the K point and the valence band maximum (VBM) at Г point. Differently, the MoSSe/WSSe vdWs heterostructure with an S–Se interface presents almost a direct bandgap with the CBM and the VBM near the K point, which is more beneficial to exciton transition. Importantly, all these MoSSe/WSSe vdWs heterostructures show a type-II band structure with the CBM and VBM resulting from the MoSSe and WSSe monolayers, which can separate the photogenerated electrons and hole. Thus, the lifetime of the photogenerated charges can be prolonged (Zhang et al., 2023).

**FIGURE 3:**
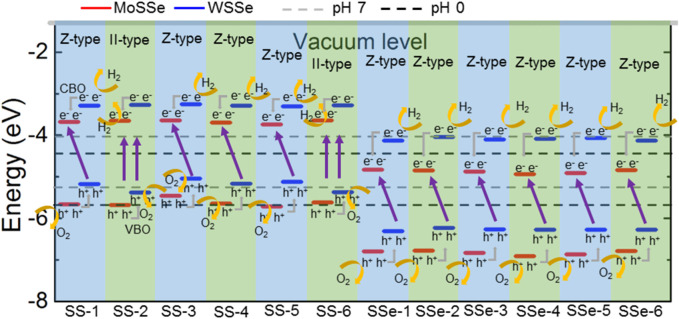
Band edge energy positions of the MoSSe/WSSe vdWs heterostructure with different stacking energy. The energy is compared with the potentials of O_2_/H_2_O and H+/H_2_ for water splitting at pH values of 0 and 7 by dash black and gray lines, respectively.

The type-II band structure of the MoSSe/WSSe vdWs heterostructure implies the oxidation and reduction reactions can be carried out separately at different layers. Figure 3 presents the band energy of the MoSSe/WSSe vdWs heterostructure with the S–S and S–Se interface comparing with the potentials of O_2_/H_2_O and H^+^/H_2_ for water splitting. One can see that the band energy of the MoSSe/WSSe vdWs heterostructure with the S–S interface is higher than that of the S–Se interface. Therefore, the MoSSe/WSSe vdWs heterostructure with the S–S and S–Se interface can be used as a photocatalyst at pH 7 and pH 0, respectively. Here, the potential of reduction and oxidation is calculated by the pH level using E_red_ = −4.44 eV + pH × 0.059 eV and E_oxd_ = −5.67 eV + pH × 0.059 eV, respectively (Ren et al., 2019b). Therefore, the reduction energy is obtained as −4.44 (−4.03) and the oxidation potential is −5.67 (−5.26) eV at pH 0 (7) for water splitting, respectively. In detail, the MoSSe/WSSe vdWs heterostructure with the SS-2 and SS-6 stacking configuration explains the traditional type-II band alignment; thus, the photogenerated electrons are excited to CBM at the MoSSe and WSSe monolayers. Then, the photogenerated electrons at the CBM of the WSSe layer further move to the CBM of the MoSSe layer by the conduction band offset (CBO). At the same time, the holes are induced at the VBM of the MoSSe and WSSe monolayers. Similarly, the photogenerated holes at the VBM of the MoSSe layer further migrate to the VBM of the WSSe layer under the valence band offset (VBO). Therefore, the oxidation and reduction reactions are induced at the WSSe and MoSSe monolayers, respectively, for water splitting at pH 7. In particular, other MoSSe/WSSe vdWs heterostructures with the S–S interface exhibit a Z-scheme photocatalyst characteristic. Because the potential of the WSSe layer is not enough to induce an oxidation reaction, the photogenerated holes at the VBM of the WSSe are more inclined to recombine with electrons in the CBM of MoSSe layer. Thus, O_2_/H_2_O and H^+^/H_2_ are conducted at CBM of the WSSe and VBM of the MoSSe layers, respectively, for water splitting as pH 7, which demonstrates an obvious photocatalytic mechanism of the Z-scheme (Ren et al., 2020b). Similarly, all the MoSSe/WSSe vdWs heterostructures with the S–Se interface present a Z-type photocatalyst for water splitting at pH 0. The oxidation and reduction reactions are explored at the MoSSe and the WSSe layers, respectively, for water splitting at pH 0, shown as in Figure 3.

**FIGURE 4:**
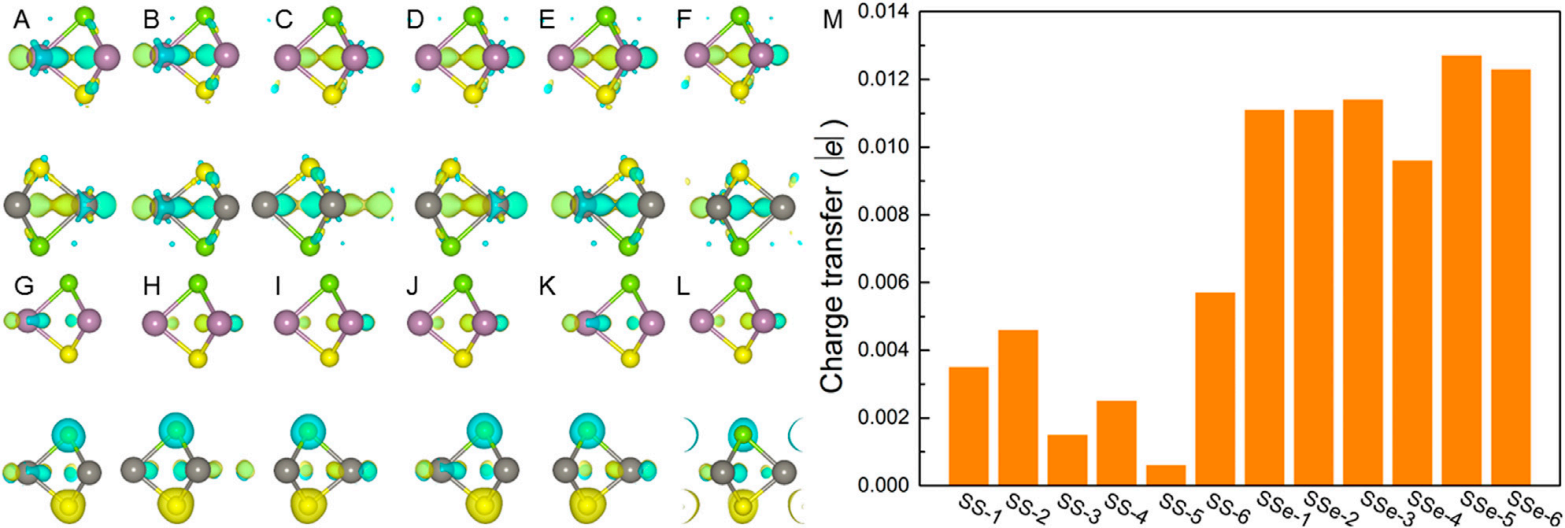
Charge density difference of the MoSSe/WSSe vdWs heterostructure with **(A)** SS-1, **(B)** SS-2, **(C)** SS-3, **(D)** SS-4, **(E)** SS-5, **(F)** SS-6, **(G)** SSe-1, **(H)** SSe-2, **(I)** SSe-3, **(J)** SSe-4, **(K)** SSe-5, and **(L)** SSe-6 stacking configurations obtained by using DS-PAW. The yellow and cyan marks demonstrate the gain and the loss of the electrons. **(M)** Calculated charge transfer from the WSSe layer to the MoSSe layer, the isosurface layer is set as 0.001 |e| for charge difference.

The charge density difference (Δ*ρ*) of these MoSSe/WSSe vdWs heterostructures is investigated, which is calculated by Eq. 2 as:

$$\Delta \rho =\rho_{\text{Heterostructure}}\;–\;\rho_{\text{MoSSe}}\;–\;\rho_{\text{WSSe}},$$ (2)

where *ρ*_Heterostructure_, *ρ*_MoSSe_, and *ρ*_WSSe_ represent the total charge of the MoSSe/WSSe heterostructure and MoSSe and WSSe monolayers, respectively. The results are demonstrated in Figures 4A–L, which explains the WSSe layer is always acting as the electron contributor, especially for the S atoms, in the MoSSe/WSSe heterostructure. Using the Bader charge-population analysis method (Sanville et al., 2007), the charge transfer in the MoSSe/WSSe vdWs heterostructure is quantified, as shown in Figure 4M. One can see that the obtained electrons of MoSSe from the WSSe layer in the MoSSe/WSSe vdWs heterostructure with the S–Se interface are higher than that of the MoSSe/WSSe vdWs heterostructure with the S–S interface, suggesting this asymmetric vdWs interface is more conducive to charge transfer.

**FIGURE 5:**
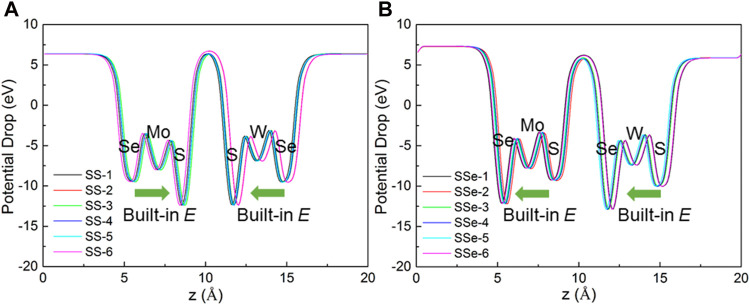
Calculated potential drop of the MoSSe/WSSe vdWs heterostructure with **(A)** S–S and **(B)** S–Se interfaces along the z direction.

Furthermore, the potential drop of the MoSSe/WSSe vdWs heterostructure with the S–S and S–Se interface is obtained in Figures 5A, B, respectively. One can see that the interlayer potential drop is almost 0 eV. Thus, the intralayer potential drop is the key to promote the separation of the photogenerated charge. In addition, the built-in electric field (*E*) of MoSSe and WSSe is also demonstrated in Figure 5. Interestingly, the direction of the built-in electric field in MoSSe and WSSe is conversely formed as the heterostructure with the S–S and S–Se interface. Thus, the overall built-in electric field is weakened in the MoSSe/WSSe vdWs heterostructure with the S–S interface, while that is enhanced in the S–Se one, which also explains the more charge transfer in the MoSSe/WSSe vdWs heterostructure with the S–Se interface. Furthermore, the potential drop of MoSSe (or WSSe) in the SS MoSSe/WSSe vdWs heterostructure is approximately 3.01 eV (or 2.815 eV), while that in the SSe MoSSe/WSSe vdWs heterostructure is calculated as approximately 2.908 eV (or 2.875 eV). One can see that the vdWs forces in the MoSSe/WSSe vdWs heterostructure almost do not change the potential difference of the original monolayers. In addition, the intrinsic dipole moment of the MoSe/WSSe system with the S–S and S–Se interface is calculated as 0.0003 |*e*|·Å and 0.0714 |*e*|·Å, respectively. Obviously, great interface asymmetry makes the material more polar, which also plays an important role in the built-in electric field to rearrange the charge in the heterostructure.

**FIGURE 6:**
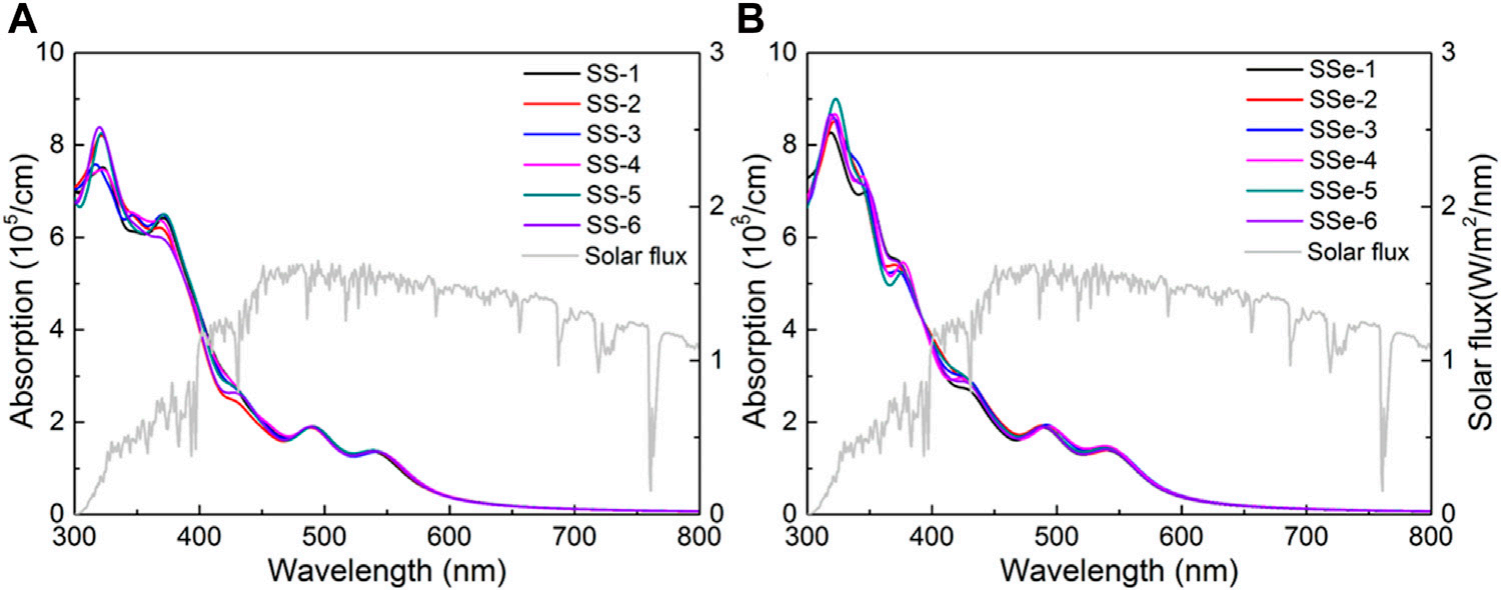
The DS-PAW obtained optical absorption spectrum potential of the MoSSe/WSSe vdWs heterostructure with **(A)** S–S and **(B)** S–Se interfaces.

Even if the MoSSe/WSSe vdWs heterostructure shows the excellent charge transfer characteristic as a Z-scheme photocatalyst, the absorption coefficient ability is also critical to be investigated. The absorption coefficient (α) of the MoSSe/WSSe vdWs heterostructure is obtained as Eq. 3 (Zhang et al., 2008):

$$\alpha \left(\omega \right)=\frac{\sqrt{2}\omega }{c}{\left\{{\left[\varepsilon_{1}^{2}\left(\omega \right)+\varepsilon_{2}^{2}\left(\omega \right)\right]}^{1/2}-\varepsilon_{1}\left(\omega \right)\right\}}^{1/2},$$ (3)

where $\varepsilon_{1}\left(\omega \right)$ and $\varepsilon_{2}\left(\omega \right)$ are used by representing the real and imaginary parts in the dielectric constant, respectively. In addition, *c* is the speed of the light. *ω* is demonstrated by the angular frequency. In addition, *ε*_2_(*ω*) can be obtained by Eq. 4 (Zhang et al., 2008)

$$\varepsilon_{2}\left(q\to O_{\hat{u}},\hbar \omega \right)=\frac{2e^{2}\pi }{\Omega \varepsilon_{0}}\sum_{k,v,c}|\langle \left.\Psi_{k}^{c}\right|{\left.\hat{u}\cdot r\left|\Psi_{k}^{v}\right.\rangle \right|}^{2}\times \delta \left(E_{k}^{c}-E_{k}^{v}-E\right),$$ (4)

where $\Psi_{k}$ , $E_{k},$ and $\hat{u}$ are selected to present the wave function, energy, and unit vector of the electric field of the incident light, respectively. $\Psi_{k}$ and $E_{k}$ mark the conduction bands and valence bands demonstrated by superscripts (*v* and *c*), respectively. The complex dielectric function is *ε(ω) = ε*_*1*_ *(ω) + iε*_*2*_ *(ω)*, and the real part ε_1_ can be obtained from ε_2_ by using the Kramers–Kronig relation. Then, the calculated HSE06 optical absorption coefficient in the visible light range is suggested in Figures 6A, B for the MoSSe/WSSe vdWs heterostructure with S–S and S–Se interface, respectively. Obviously, all these MoSSe/WSSe vdWs heterostructures possess novel optical performance. For the MoSSe/WSSe vdWs heterostructure with the S–S interface, the SS-6 heterostructure shows an absorption peak at approximately 8.42 × 10^5^ cm^−1^ at the wavelength of 320 nm. The SS-5 MoSSe/WSSe vdWs heterostructures present an absorption peak at approximately 6.51 × 10^5^ cm^−1^ at the wavelength of 372 nm, and the absorption peak also exists near the wavelength of approximately 490 nm and 543 nm, as shown in Figure 6A. For the MoSSe/WSSe vdWs heterostructure with the S–Se interface, more excellent light absorption properties are demonstrated. The SSe-6 MoSSe/WSSe vdWs heterostructures possess an absorption peak at approximately 8.96 × 10^5^ cm^−1^ with the wavelength of 323 nm. Then, the SSe-2 MoSSe/WSSe vdWs heterostructures have superior absorption properties in the visible range of approximately 5.46 × 10^5^ cm^−1^ with the wavelength of 378 nm. An additional absorption peak of the MoSSe/WSSe vdWs heterostructures with the S–Se interface was also obtained near the wavelength of 490 nm and 543 nm, as shown in Figure 6B. The obtained light absorption properties of the MoSSe/WSSe vdWs heterostructures with different stacking configurations are higher than that of the reported 2D heterostructure using as a photocatalyst for water splitting, such as AlN/Zr_2_CO_2_ (about 3.97 × 10^5^ cm^−1^) (Ren et al., 2021b), CdO/Arsenene (about 8.47 × 10^4^ cm^−1^) (Ren et al., 2021c), and MoSSe/Mg(OH)_2_ (about 1.43 × 10^5^ cm^−1^) (Lou et al., 2021).

## Conclusion

In this work, the MoSSe/WSSe heterostructure is constructed by the S–S and S–Se interface; the band structure and the optical performances are then systematically investigated by density functional theory. Interestingly, all these MoSSe/WSSe heterostructures are formed by vdWs interactions, and the structural parameters also show significant differences. The electronic performance of the MoSSe/WSSe vdWs heterostructure explains the intrinsic semiconductor properties which are not changed by the stacking configuration. Although the band alignment presents obvious dependence that both traditional type-II band and Z-scheme structures can be tuned. Furthermore, the more charge transfer is addressed in the MoSSe/WSSe vdWs heterostructure with the S–Se interface comparing with the S–S interface is contributed from the enhanced built-in electric field, and all the stacked MoSSe/WSSe vdWs heterostructures possess excellent light absorption capacity. The results show the MoSSe/WSSe vdWs heterostructure can be used as a tunable photocatalyst for water splitting, and the stacking method is an efficient method to induce the Z-type photocatalytic mechanism.

## Data Availability

The raw data supporting the conclusion of this article will be made available by the authors, without undue reservation.
