# Supplementary material for: Stacking engineering induced Z-scheme MoSSe/WSSe heterostructure for photocatalytic water splitting
Source: Front Chem. 2024 Jun 28;12:1425306. doi: 10.3389/fchem.2024.1425306 (PMC11239575; doi:10.3389/fchem.2024.1425306)
Supplement: Supplementary file 1 [file DataSheet1.pdf]

Supporting Information

Stacking Engineering Induced Z-scheme MoSSe/WSSe Heterostructure for Photocatalytic Water Splitting

Guoliang Fan[1], Zongfa Liu[2], Zhen Ma[3], Kai Ren[4,5,*] and Zhen Cui[6] and and Weihua Mu[*,7]

[1]Department of Automotive and Mechanical Engineering, Anhui Communications Vocational & Technical College, Hefei 230051, China

[2]School of Automotive Engineering, Weifang Vocational College, Weifang 262737, China

[3]School of Agricultural Engineering, Jiangsu University, Zhenjiang 212013, China

[4]School of Mechanical and Electronic Engineering, Nanjing Forestry University, Nanjing 210037, China

[5]Medical Oncology, Luoyang Central Hospital, Luoyang 471099, China

[6]School of Automation and Information Engineering, Xi'an University of Technology, Xi'an, Shaanxi 710048, China

[7]Wenzhou Institute, University of Chinese Academy of Sciences, Wenzhou, 325000, China

Corresponding Author: kairen@njfu.edu.cn (Kai Ren), Weihua Mu (E-mail: muwh@ucas.ac.cn)

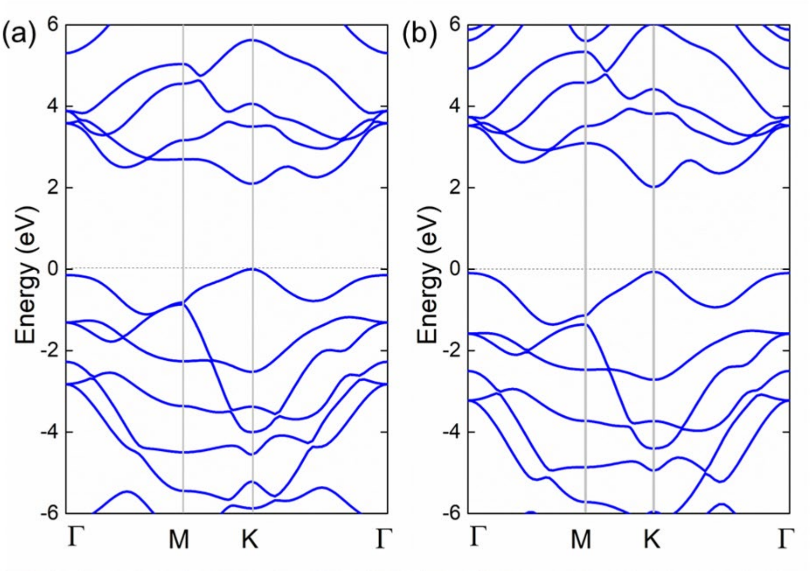

Figure S1. The calculated band structure of the (a) MoSSe and (b) WSSe monolayers using HSE06 method.

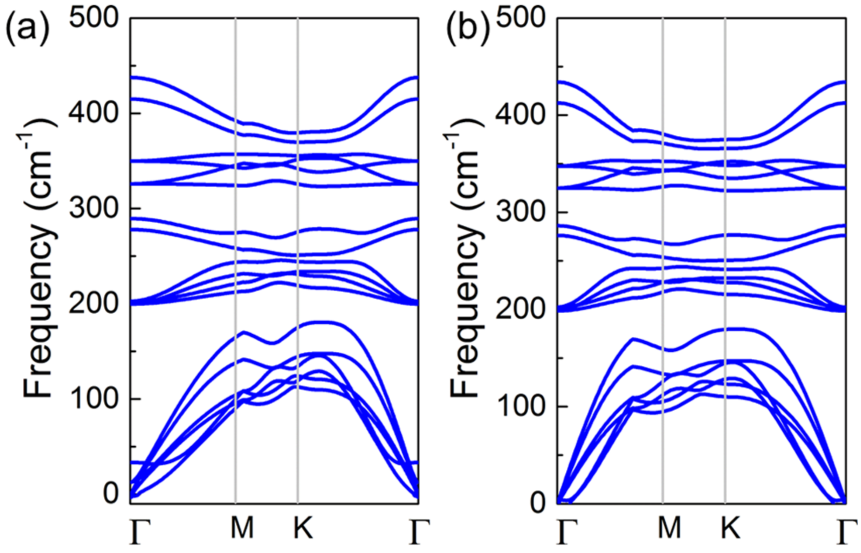

Figure S1. The calculated phonon dispersions of MoSSe/WSSe vdWs heterostrutcure with (a) SS-1 and (b) SSe-1 stacking configurations.
